# Effect of sugar on male *Anopheles gambiae *mating performance, as modified by temperature, space, and body size

**DOI:** 10.1186/1756-3305-2-19

**Published:** 2009-04-22

**Authors:** Richard E Gary, James W Cannon, Woodbridge A Foster

**Affiliations:** 1 Department of Entomology, Aronoff Laboratory, 318 West 12th Avenue, The Ohio State University, Columbus, Ohio, USA. 43210-1242; 2 Zoonotic Disease Program, Ohio Department of Health, 8955 E. Main Street, Reynoldsburg, Ohio, USA. 43068

## Abstract

**Background:**

*Anopheles gambiae *plant-sugar feeding was thought to be rare and physiologically optional. Unlike adult females, males have no alternative source of energy and soon die with only water, yet they might be competent to inseminate all females within their brief lifespan. This study was designed to detect sugar's effect, if any, on male performance.

**Methods:**

Males with and without 20% sucrose were evaluated at two body sizes and two temperatures, 23° and 27°C. Survival was recorded twice daily, and sexual behaviour was recorded each night after adult emergence. Insemination at a 2:1 male:female ratio was examined in three cage sizes, including walk-in mesocosms.

**Results:**

Without sugar, males of both sizes lived longer at 23° than 27°C, and large males lived longer at each temperature. Survival of large males at low temperature averaged 3.7 days, small males at high temperature, 1.9 days. With sugar, males in all four treatments suffered minimal mortality. With sugar, in small cages, large males at 27°C matured most rapidly. A few erected fibrillae and inseminated females on night 1. On night 2, maximal proportions erected fibrillae and swarmed, and over one-third of females became inseminated. Small sugar-fed males at 23°C matured most slowly but had achieved nearly maximal levels of swarming by night 3. By night 5, small males had inseminated more than half the females, and large males had inseminated nearly all of them. Without sugar, large males progressed similarly during the first two nights. On night 3, however, the proportion erecting fibrillae and swarming declined precipitously at 27°C, and to a lesser degree at 23°C. Cumulative insemination never reached high levels. Small males never achieved high levels of fibrillar erection or swarming and inseminated few females, even at 23°C. In larger cages and under more semi-natural conditions, regardless of body size, without sugar male insemination capacity was virtually nonexistent.

**Conclusion:**

Under some conditions, a limited number of sugar-deprived males can survive long enough to inseminate females. However, in nature males that cannot obtain sugar at frequent intervals will not be competitive with those that can, suggesting that male performance is closely tied to plant communities.

## Background

Sugar from plant juices is the only food resource of adult male mosquitoes. Although both sexes feed on sugar to build energy reserves [1], males probably feed on it more frequently because 1) females contribute to their maternal reserve by blood feeding [2-4], and 2) males are relatively poor at building metabolic reserves from sugar [2,5-7]. Males that emerge with low teneral reserves are especially vulnerable to starvation if they are unable to feed on sugar soon after emergence [8-10].

Low teneral energy reserves typically are associated with crowded larval conditions because of the increased competition for limited food. However, some species inherently build a very small teneral reserve, even when larval conditions are ideal. One such species is *Anopheles gambiae *Giles *sensu stricto *[4], the principal African vector of the malaria pathogen *Plasmodium*. Both sexes emerge with little available energy, and both sexes are strongly attracted to nectar-related volatiles when newly emerged and often prefer them to host-related volatiles under laboratory conditions [10]. This preference suggests that sugar feeding is an early priority, probably due to the risk of starvation. Despite this vulnerability to energy depletion, *An. gambiae *is believed by researchers to feed on sugar in nature only facultatively and rarely [11-14]. McCrae [13] suggested that sugar sources in tropical Africa are too restricted in time, place, or attractiveness to imply more than facultative feeding. The solution to the energy-deficit problem for females is to feed more frequently on blood, mostly from humans [12,14-17], which may help to explain the unusual importance of this mosquito in malaria transmission. Males, however, cannot take blood, so they would be confronted with a more severe problem than females, if nectar sources are scarce. They emerge from the same breeding sites as females, form outdoor mating swarms in the vicinity of human habitations [18-20], where females get their blood meals. Many males rest inside houses along with females and may mate with them there, as well [21]. It seems unlikely that males would share the females' habitat but travel unknown distances to find the rare sources of sugar. Therefore, if most females become inseminated even where natural sugar is generally scarce, then many males must be able to survive long enough to swarm and mate without it. Possibly, they are adapted to this situation by a fast reproductive maturation and frequent early mating, in the likely event of a short lifespan. This is consonant with the report that *An. gambiae *males achieve maximum mating capacity as early as 3 days after emergence [22]. Additionally, females are more likely to oviposit when mated with 2–3 day old adult males than with older males [23].

Therefore, at issue is whether males lacking plant sugar can nonetheless perform adequately. If this is not the case, perhaps *An. gambiae *populations can be reduced indirectly, by depriving males of their plant hosts. To address this question, we observed and tested the effect of sugar availability on antennal fibrillar erection, swarming flight, and ability to inseminate females, during and after successive post-emergence evening crepuscular periods. Large and small males were evaluated at two temperatures and in three enclosure sizes.

## Methods

### Maintenance and rearing

*An. gambiae *used in all experiments were of the Suakoko strain, molecular form M, established by M. Coluzzi in 1987 from material originating in Suakoko, Bong, Liberia. Colony adults were maintained in 86-L cages supplied with honey-soaked sponges, water, and periodic human blood meals. Blood feeding was conducted in accordance with The Ohio State University's Biosafety protocol No. 2005R0020 and Biomedical IRB protocol No. 200440193, FWA No. 00006378. Oviposition cups were placed with caged adults 2 days after each blood meal, and eggs were collected the following day. The laboratory conditions for the colony were 27 ± 1°C, 80 ± 5% RH, and 13:11 (L:D), with 75-minute gradual crepuscular transitions between photophase and scotophase. Laboratory temperatures did not fluctuate as they would under natural field conditions. Instead, two different laboratory temperatures (23 ± 1°C and 27 ± 1°C) were used in the experiments, each of which occurs within the natural temperature range at times when breeding is common in equatorial Africa. These two temperatures allowed for different speeds of energy-reserve consumption and sexual maturation, in case survival rate and maturation rate do not offset one another in a strictly proportional manner. Mosquitoes to be studied at 23°C were transferred to this temperature on the day following pupation.

Larvae for both the colony and experiments were reared as follows: Eggs were disinfected with 0.05% sodium hypochlorite solution and hatched overnight in flat enamel-coated pans of aged tap water. To produce adults of natural extremes of body size, first-instar larvae were placed, 100 each (for large-bodied) or 1000 each (for small-bodied), in 22.8 × 33.0-cm aluminium pans with 450 ml of aged tap water. The larvae were fed a standard diet of pulverized TetraMin^® ^fish flakes, following a daily schedule that provided 0.5 mg (for large) or 0.01 mg (for small) of food per larva per day. Pupae were collected daily, segregated in separate 38-L cages (internal dimensions ~45 × 29 × 29 cm, h, w, d respectively) according to rearing regime, and supplied with water wicks. Males and females emerging overnight were separated the following morning, by which time no insemination had occurred. All experimental mosquitoes had constant access to plain water throughout the experiments, regardless of enclosure size or sugar-treatment group.

To establish that rearing regimens produced groups of adults with significantly different body sizes, samples of adults were set aside. One wing from each adult was removed, mounted on a slide and measured from the distal end of the alula to the wing tip to the nearest 0.0001 mm using an Auto-Montage digital imaging microscope (Syncroscopy, Frederick, Maryland, USA).

### Survival

Newly emerged males were divided into three groups of approximately 50 individuals. Each group was placed into an 80-L acrylic cage (internal dimensions ~45 × 51 × 35 cm, h, w, d, respectively) supplied with two water-soaked cotton wicks. The first group received no sugar. The second group was supplied with two additional cotton wicks soaked with 20% sucrose and allowed to sugar feed ad lib. The third group was given a single meal of 20% sucrose before they were placed in the experimental cage. To accomplish this, the group of males was placed in a screen-lidded 500 ml plastic cup, lined with a paper towel soaked with 20% sucrose. After mosquitoes were allowed to feed for 10 minutes, by which time all were engorged and had ceased feeding, they were transferred to the experimental cages. Survival was recorded twice daily. This experiment was repeated in two replicates each, with both large and small males, each size at 23°C and at 27°C. The experiment was terminated on day 6.

### Reproductive behaviour

After eclosion, males were transferred, 20 each, into two 38-L cages supplied with four water-soaked cotton wicks each. One cage also was supplied with four wicks soaked with 20% sucrose. A 15-watt red lamp provided ambient light during the crepuscular period and onset of scotophase, when the number of males the number of males swarming was recorded at 5-minute intervals through the crepuscular period and first hour of darkness. The number of males with extended antennal fibrillae was also recorded prior to and after the flight period. It was not possible to record this independently during the swarming period. Although the activity of males approximated the distinctive, tightly looping flight described by Charlwood and Jones [24] and interpreted as swarming, the observation cages were too small to determine whether every airbourne male was involved in swarming behaviour. Therefore, for the purposes of this experiment, the number of males in flight (total number of males, less the number at rest), once antennal fibrillae were extended, was considered to be the number swarming. This experiment was replicated four times each, at 23°C and at 27°C, within each size class. It was terminated after 4 nights.

### Insemination success

Three separate experiments were conducted to determine the effect of sugar on male insemination success. Three sizes of enclosures were used, to allow either for greater random contact between males and females or for more space for swarming. In all experiments, the female:male ratio was 1:2, to ensure sufficient insemination data for robust statistical analysis in the groups of males expected to perform poorly and die quickly, due to lack of sugar. This consideration outweighed the alternative one, which is to provide males with more opportunities to encounter uninseminated females. In nature, the operational sex ratio is typically skewed strongly toward males. In the first experiment, 60 newly emerged large males were placed into each of two 38-L acrylic cages supplied with four water-soaked cotton wicks each. One cage was also supplied with four wicks soaked with 20% sucrose. Thirty sugar-fed virgin females, 3–5 days old, were placed into each cage at least 4 hours prior to onset of the crepuscular period. The females were allowed to feed from emergence until they were put into experimental cages. To determine cumulative insemination rate over several days, different groups of females in separate cages were left with the same newly emerged males for 1–5 consecutive nights, or until most males were dead, after which females were removed, dissected in saline, the spermatheca removed and placed under a coverslip on a glass slide, and the spermatheca checked visually for the presence of sperm at 100 and 450× magnification. This experiment was replicated four times at each time interval, both at 23°C and at 27°C, and with both large and small males. At 23°C, groups of females were left with males for 1,2,3 or 5 consecutive nights before dissection. At 27°C, the time intervals were 1,2, or 5 consecutive nights.

In the second experiment, the procedure was the same except that 80-L acrylic cages were used, to provide more space for swarming, and the experiment was replicated four times at 27°C with large males only. To determine cumulative insemination rate, different groups of females in separate cages were left with newly emerged males for either 1, 2 or 5 consecutive nights, or until most males were dead, after which females were removed and dissected to check for the presence of sperm in the spermathecae.

In the third experiment, 100 males were released into each of two greenhouse mesocosms, described below, the morning after emergence. One mesocosm was supplied with 10 hanging yellow cellulose sponges (~10 × 6 × 1 cm) covered with honey. Honey was used instead of sucrose so that mosquitoes could locate it easily from a distance, by orienting to its volatiles. In each of three replicates, the two treatments groups were alternated between mesocosms to control for possible local environmental effects. Each morning, 50 5-to-6-day-old females were released from 7-L acrylic cages supplied with cotton wicks soaked with 20% sucrose, where they had acclimated to the mesocosm environment since emergence. Most were recaptured the following morning by backpack aspirator (for host-attracted females) and mouth aspirator (for resting females), prior to the release of the next group of females. Recovery of every released female on each subsequent morning was not possible, so an average of 6 females captured on subsequent mornings may have spent 2 or more nights in the mesocosm. However, this proved to be equally probable in both treatment groups, so systematic errors were not a concern. Captured females were frozen, then dissected to determine daily insemination success. An attempt was made to count resting males each day to determine survivorship. After night 4, all mosquitoes were removed for a final accounting of male survival and female insemination.

The mesocosms used in this experiment were screen enclosures built within two separate rooms located in a greenhouse facility. Each enclosure was a wooden frame, covered with mosquito netting. Both mesocosms had the same dimensions of 2.84 × 3.63 × 2.08 m (w, l, h) (= 21.4 m^3^). A two-compartment antechamber served as a safe room to prevent mosquito escape. The mesocosms were illuminated primarily by direct and indirect sunlight, although to simulate an equatorial photoperiod (12:12, L:D), a portion of the winter morning was lighted by an incandescent light on a timer. The minimum temperature was maintained by wall radiators heated by steam and regulated by a sheltered, centrally positioned thermostat. Temperatures fluctuated from 23° to 30°C. Humidity cycled between 50% and 90% RH by mist-spraying devices and humidifiers. Walls of concrete cinder blocks, holding beds of wet sand, stood along one wall of each mesocosm to provide resting sites for the mosquitoes and increase moist surface areas that helped to stabilize humidity and provide water for drinking. At dusk males engaged in looping flights in loose aggregations in a distinctive place, indicating that a behaviour akin to natural swarming occurred. However, the swarm did not form over a classic marker, and swarming activity was not quantified.

### Statistical analysis

Survivorship data were analyzed by a Kruskal-Wallis test for multiple treatments. For the purposes of this analysis, the day of death for males with ad-lib sugar was recorded as time of censoring (discontinuation of the experiment). Post-hoc multiple comparisons between treatments and against a control were performed using Statistica [25], applying the method of Siegel and Castellan [26]. Other results (body-size difference; sugar effects on fibrillar erection, swarming, and insemination) were analyzed by Student's *t *test and Chi-square test. Differences among replicates of experiments were found to be trivial, so data were combined prior to analysis.

## Results

### Size

Wing-length measurements for males reared at densities of 100 per pan ( = 2.87 ± 0.02 mm) and 1000 per pan ( = 2.61 ± 0.02 mm) demonstrated that rearing procedures resulted in two significantly different body-size classes (*t *test, *P *< 0.0001). Based on field measurements [27,28], these size classes are representative of natural extremes of adult size.

### Survival

**Figure 1:**
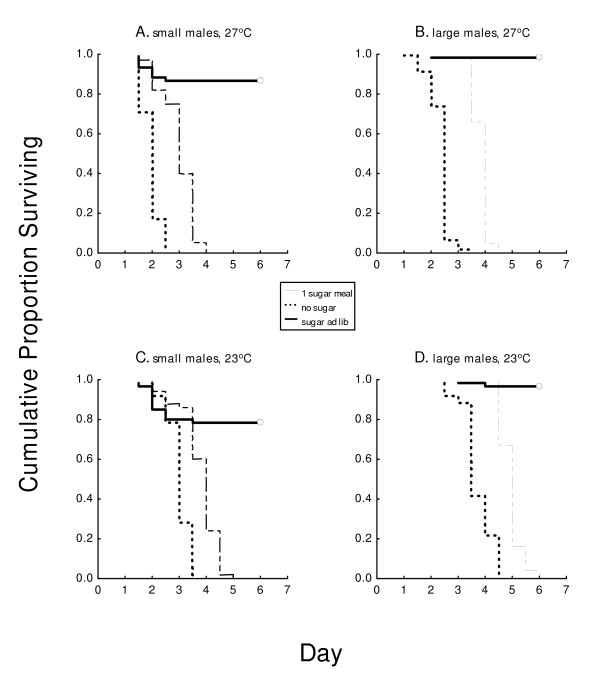
**Survival of small (A, C) and large (B, D) males at 27°C (top) and 23°C (bottom) given 1 sugar meal (20% sucrose) the evening after emergence, given sugar ad lib, or given water only (all had water), in 80-L cages**. Open circles denote censored data. (~50 males in each treatment, each replicated 2 times; see Table 1).

Without sucrose, at each temperature large males lived significantly longer than small males, and males at 23°C lived longer than their same-size counterparts at 27°C. For example, at 23°C large males lived significantly longer ( = 3.72 days) than small males ( = 2.99 days) (*P *< 0.0001) and longer than both large and small males at 27°C ( = 2.36 days and 1.94 days, respectively) (*P *< 0.0001) (Table 1, Figure 1). At both low and high temperatures, a single 20% sucrose meal significantly increased the lives of large males by slightly more than 1 day ( = 4.94 days and 3.84 days, respectively) (*P *< 0.0001). Among small males, a significant extension of life afforded by a single sucrose meal also was slightly more than 1 day at 27°C ( = 3.15 days) and slightly less than 1 day at 23°C ( = 3.77 days) (*P *< 0.007). In all survival experiments, the majority of males were dead by 3.5 days. Without sugar, the longest survival time was 4.5 days, achieved by a few large males at 23°C (Figure 1).

**Table 1:** Effect of sugar feeding on survival times of large and small virgin male *An. gambiae *at 2 temperatures.

	Median days survived; Mean days survived* ± SEM; (N)	
	
Treatment	Large Males	Small Males
23°C		
no sugar	3.5; 3.72 ± 0.073a, b; (97)	3.0; 2.99 ± 0.056b, d; (101)
1 meal 20% sucrose	5.0; 4.94 ± 0.039e; (100)	4.0; 3.77 ± 0.070a; (100)
20% sucrose ad lib**	> 6.0 f; (100)	> 6.0 f; (99)
27°C		
no sugar	2.5; 2.36 ± 0.031c, d; (120)	2.0; 1.94 ± 0.034c; (100)
1 meal 20% sucrose	4.0; 3.84 ± 0.033a; (103)	3.0; 3.15 ± 0.062b; (100)
20% sucrose ad lib**	> 6.0 f; (100)	> 6.0 f; (100)

* Means with different letters are significantly different (P < 0.05) after post-hoc multiple comparisons between treatments and against a control (method of Siegel and Castellan 1988) in a Kruskal-Wallis rank test.

**Data were censored at 6 days.

### Fibrillar erection and swarming behaviour

**Figure 2:**
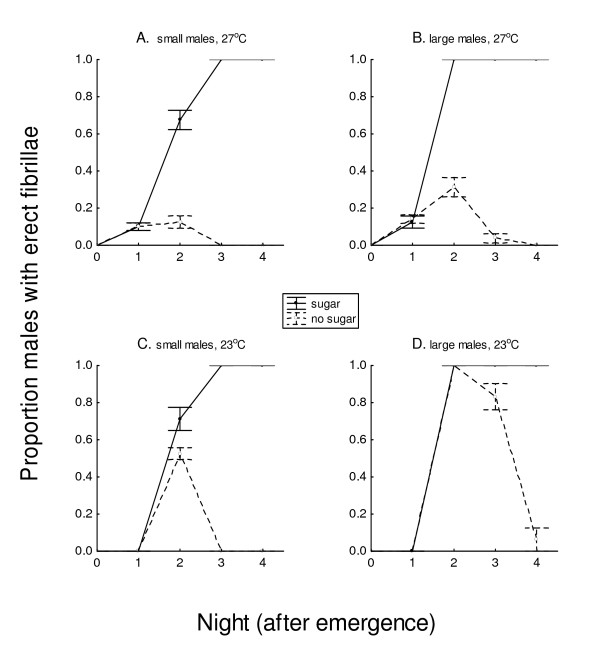
**Extension of antennal fibrillae (maximum proportion ± SEM), according to presence or absence of available sugar (20% sucrose) on small (A, C) and large (B, D) males on each night after emergence, at 27°C (A, B) or 23°C (C, D), in 80-L cages**. More males with sugar access erected their fibrillae than males without sugar (*P *= 0.05), starting from night 2 (A, B, C) or night 3 (D) onward. (20 males in each treatment, each replicated 4 times).

On the first night (night 1, 24 hours after emergence), a small proportion of both large and small males had erect fibrillae at 27°C at some time during the crepuscular period or early scotophase (Figure 2a,b), whether or not sugar was available, but most fibrillae returned to a recumbent position within 10 minutes during the crepuscular period. Fibrillae of males at 23°C remained recumbent the first night after emergence (Figure 2c,d). By the second night, up to 100% of sugar-fed large males at both temperatures extended their antennal fibrillae (Figure 2b,d), which occurred just prior to the beginning of scotophase and lasted until just after swarming (ca 1.0 hour). On night 2, at 27°C, only 20% of remaining males without sugar had erect fibrillae (Figure 2), which were sustained less than 40 minutes. In contrast, at 23°C 100% of large males both with and without access to sugar had fibrillar erections on night 2. On night 3 at 27°C (Figure 2) and night 4 at 23°C (Figure 2), there were no erect fibrillae among starved males, and none lived through the night.

**Figure 3:**
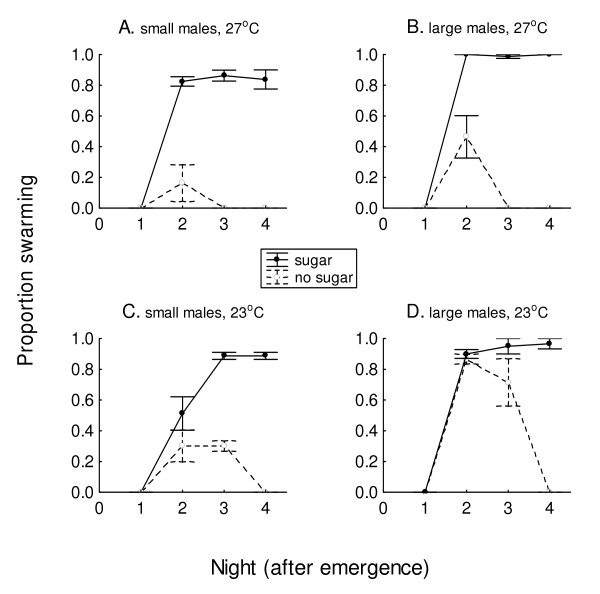
**Swarming (maximum proportion ± SEM), according to effect of presence or absence of available sugar (20% sucrose) on small (A, C) and large (B, D) males on each night after emergence, at 27°C (A, B) or 23°C (C, D), in 80-L cages**. More males with sugar access swarmed than males without (*P *= 0.05), starting from night 2 (A, B, C) or night 3 (D) onward. (20 males in each treatment, each replicated 4 times).

In general, sugar was necessary for swarming in both large and small males at 27°C, but not at 23°C (Figure 3). Swarming was first observed the second night after emergence. When sugar was available, swarming was significantly more common at 27°C in males of both sizes (*X*^2 ^= 73.01 and 87.13, respectively, *P *< 0.05 both) and at 23°C in small males (*X*^2 ^= 9.13, *P *< 0.05) than when sugar was not available. That was not the case for large males at 23°C (*X*^2 ^= 0.54, *P *> 0.05); those with and without sugar swarmed in similar numbers. By night 3, when sugar was present, a higher proportion of large and small males swarmed at both 23°C (*X*^2 ^= 20.01 and 71.54 respectively, *P *< 0.05 both) and 27°C (*X*^2 ^= 200 and 151.65 respectively, *P *< 0.05 both). Without sugar, swarming activity by males of both sizes ceased after the second evening at 27°C, and after the third evening at 23°C (Figure 3). With sugar available, swarming activity was near 100% for both body sizes, starting on night 2 at 27°C and on night 3 at 23°C.

### Insemination success

**Figure 4:**
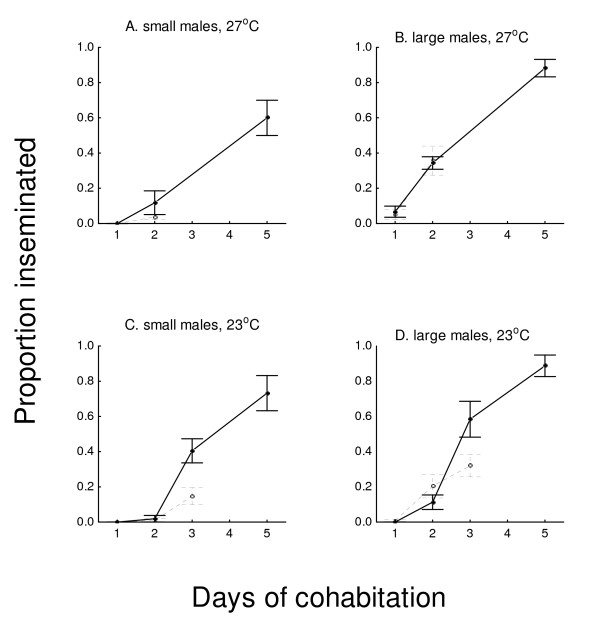
**Insemination (proportion of females inseminated ± SEM) according to effect of presence or absence of available sugar (20% sucrose) on small (A, C) and large (B, D) males, at 27°C (A, B) or 23°C (C, D), after 1–5 days of cohabitation with females in 38-L cages, starting after the males' emergence**. (60 males + 30 females per time period, each replicated 4 times).

In 38-L cages, when sugar was present, cumulative insemination rates increased over 5 days from 0 to 88% (large males at both temperatures), to 60% (small males at 27°C), and to 73% (small males at 23°C) (Figure 4). In the absence of sugar, insemination increased similarly to those with sugar over the first two nights at 27°C and over the first three nights at 23°C. After two nights of mating opportunity, sugar still had no significant effect in any treatment group, except among small males held at 27°C, in which sugar-deprived males inseminated slightly fewer females (4%) than males with sugar (12%) (*X*^2 ^= 4.60, *P *< 0.05). By day 3, there were no surviving males at 27°C in sugar-deprived groups. However, at 23°C, both large and small sugar-deprived males were still alive and had inseminated females (32% and 15% respectively). This was still significantly less than insemination rates of those with sugar (58% and 41%, respectively) (*X*^2 ^= 14.17 and 16.58 respectively, both *P *< 0.05), and no inseminations occurred beyond night 3 among sugar-deprived males.

**Figure 5:**
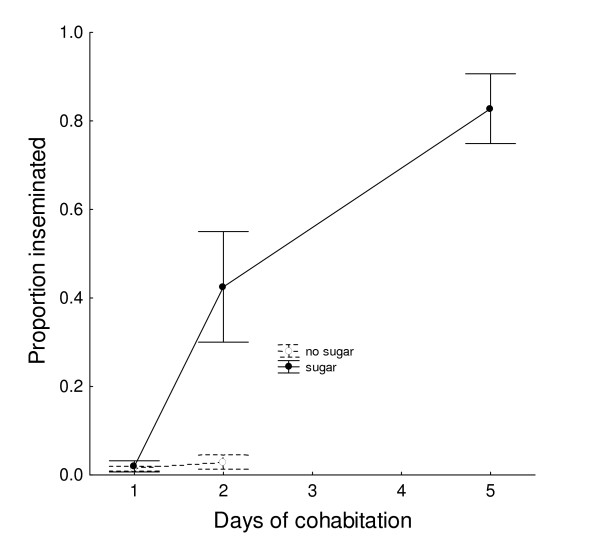
**Insemination (proportion of females inseminated ± SEM) according to effect of presence or absence of available sugar (20% sucrose) on large males at 27°C after 1–5 days of cohabitation with females in 80-L cages, starting after the males' emergence**. (60 males + 30 females per time period, each replicated 4 times).

In 80-L cages at 27°C, the large males with access to sugar produced results similar to those above. Unlike the experiment above, however, sugar-deprived males had very low rates of insemination after the first (1%) and second (3%) nights, and no inseminations after that (Figure 5). Males with sugar had much higher insemination rates after two days (43%) (*X*^2 ^= 44.68, *P *< 0.05). After five nights, males with sugar had inseminated 82% of the females.

**Figure 6:**
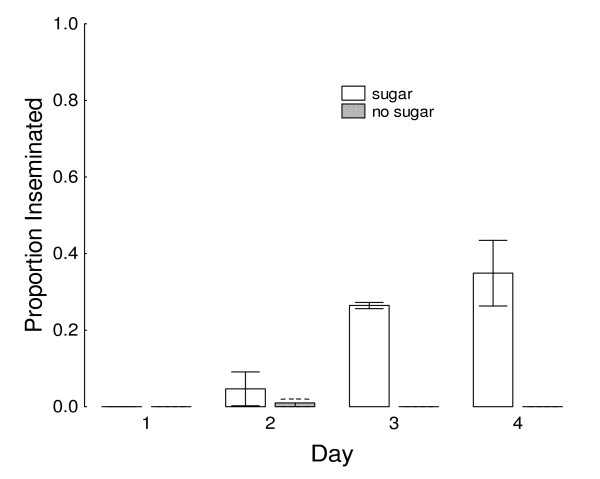
**Insemination (proportion of 50 females inseminated ± SEM) by the same 100 large males per 24 hours, according to effect of presence or absence of available sugar (undiluted honey) after each of four 1-day periods of cohabitation with new females in a greenhouse mesocosm, starting after the males' emergence**. Values not cumulative. Temperatures fluctuated daily between 23°C and 30°C. (50 females introduced on one day and removed the next day in each treatment on four consecutive days; replicated 3 times).

In the mesocosms, results were similar to the 80-L cage experiment above, except that very few inseminations occurred before night 3 (Figure 6). (Unlike the cage experiment, the mesocosm results are based on only single nights of insemination for one cohort of males, so their total performance is based the compilation inseminations of all four nights.) The insemination rate rose substantially on night 3 if honey was available (26.5%) but not if honey was withheld (0%) (*P *< 0.05). Daily insemination rates remained high after night 3 when honey was available, but most males without honey were dead by night 3. The average cumulative insemination rates were 71% with honey and 1% without honey. Daily counts of resting males suggest that, in each replicate, there was low survivorship the first night, whether or not honey was present ( = 41.3% and  = 42.3%, *P *= 0.51). After the first night, survival remained high in the room with honey while declining in the honey-deprived room. After the 3^rd ^night, 38% of males remained alive in the honey-supplied room and 3.6% remained alive in the honey-deprived room.

## Discussion

In nature, male *An. gambiae *in many locations form daily mating swarms that last for about 0.5 hours at dusk [19,20], during which time females entering the swarm are inseminated. Just prior to taking flight, the antennal fibrillae of males in the laboratory become erect and remain so until the end of swarming [22]. In the present study, mature male *An. gambiae *performed similarly, after completing a period of maturation required to exhibit these behaviours. Because swarms have not been found in all sites where this species has been investigated, it remains possible that an alternative aggregating site or mechanism for mate-finding exists. The observations reported here, however, indicate that this Liberian strain mates in the air while the males are engaged in frenzied looping flights, at least within the restricted spaces supplied. Whether the swarming observed in our enclosures is the equivalent of what often occurs in the field, either within houses or outside, cannot be answered at this time.

Male anophelines of other species rarely swarm in the field before the second night after emergence [29,30]. We likewise found that very few male *An. gambiae *erect their antennal fibrillae or swarm before the second night after emergence. By then, their ability to do so without sugar feeding was associated with larger body size and lower temperature. Presumably, the larger males had higher teneral reserves to support survival and sexual activity [4], and the lower temperature (23°C) slowed the depletion of those reserves without retarding reproductive maturation to such a degree. However, by the third day, the effects of sugar deprivation were evident in the reduced performance of these behaviours, no matter the size class or temperature.

Daily insemination ability of males having sugar increased dramatically on the third night after emergence in the semi-natural conditions of the mesocosms. Other studies of sugar-fed *Anopheles *spp. have demonstrated that insemination ability peaks at three days [22,31,32] or even seven days after emergence [33]. Females are more likely to oviposit when inseminated by 2–3-day-old males than by older males [23]. Therefore, sugar-deprived males, which often can survive long enough to exhibit appropriate mating behaviours, conceivably might be sufficiently competent to inseminate all females of the same cohort. However, we found that in large cages and in mesocosms, the mating capacity of sugar-deprived males was very low, often nonexistent, and never reached that of sugar-fed males. The rate of mortality in the mesocosms, though steep at the outset of the experiment, was only slightly higher than that in the cages after 3 days and probably did not contribute appreciably to the exceptionally low insemination rates among mesocosm males lacking honey. Lack of energy to become sexually active and to locate females seems more likely.

Only in smaller cages was the insemination capacity of sugar-deprived males as high as that of sugar-fed males, and then only for the first two days and only in large-bodied males. As well as having a larger energy reserve for survival, maturation, and behavioural activity, the larger males may have been more effective in obtaining mates, as observed in some studies of anophelines [34,35]. In the field, energy demands are expected to be higher than in the laboratory. This species emerges with low teneral reserves [4], and male mosquitoes in general are poor at building metabolic reserves. So sugar feeding is likely to be an early priority for both sexes [1,10,36], and males would not travel far to obtain sugar. This is particularly true of small males, which make up a substantial proportion of some natural populations and, if they succeed in joining a swarm, appear to mate just as successfully in the field as larger males [28]. Without sugar, a vast majority of small males in the present study survived less than three days and had virtually no insemination success. Therefore, their likelihood of joining a swarm is probably greatly reduced. We conclude that the diminished insemination ability of males without sugar is mediated by a combination of retarded sexual behaviour and reduced survival.

While highly artificial, the small-cage experiment with large males probably represents the upper limit of what is possible under the most favourable conditions, given that the Suakoko strain is adapted to small cages. The large cage and mesocosm experiments, by contrast, may indicate what would occur most often in nature, unless mating commonly is achieved indoors by the M form of *An. gambiae *or is a tactic adopted by nutritionally compromised individuals [21]. Therefore, while some sugar-deprived males can inseminate females, especially in confined spaces within the first two nights after emergence, they may seldom do so in nature. Alternatively, conceivably a non-cage-adapted mosquito might have performed better in these large spaces. Either way, the absence of sugar severely restricts mating potential, and males that can find sugar in nature will have a very large competitive advantage.

Male dependence on plants offers some opportunities for intervention of the malaria-vector population. For the introduction of sterile or genetically altered males, providing them with sugar meals prior to release may give them a critical competitive advantage over wild males. This may be necessary just to offset an inferior ability of laboratory-adapted males to locate natural sources of sugar. Another prospect is to alter the host plants and their communities. If the plant hosts of *An. gambiae *are limited to a few dispensable species, selective removal of these plants in the vicinity of human habitations, and their replacement by innocuous or beneficial species, may be sufficient to cause a significant proportion of females to suffer delayed insemination or to remain uninseminated, resulting in delayed or reduced oviposition, reduced biting frequency, and population suppression. The net effect would be a lower vectorial capacity and a smaller target for sterile or genetically altered males. Similarly, preferred plants may be made toxic, causing severely reduced survival of one or both sexes and leading to local population elimination, as has been demonstrated for *An. sergentii *in Israel [37,38].

## Conclusion

We conclude that *An. gambiae *males require early and frequent access to nectar or other sugar sources in their habitat in order to become sexually mature and engage in repeated mating activity. In contrast to McCrae's [13] conclusion that plant-sugar sources are too restricted to be of any importance to this species, we expect that sugar sources are readily available in areas where this species thrives. If females feed restrictively on sugar [14], it is not due to a lack of available nectar sources. Males, and also females, can survive by feeding on the fluids of plants commonly found around villages and rural houses [39-41].

## Competing interests

The authors declare that they have no competing interests.

## Authors' contributions

RG designed and conducted all cage experiments and performed statistical analyses of the results, JC constructed the mesocosms, and JC and RG collaboratively designed and conducted the mesocosm experiments. WF developed the conceptual background, constructed the cages, assisted with experimental design and with construction of the mesocosms, and obtained partial funding for the project. All authors contributed to preparation of the manuscript.
